# Supplementary material for: Time-series transcriptome analysis identified differentially expressed genes in broiler chicken infected with mixed Eimeria species
Source: Front Genet. 2022 Aug 8;13:886781. doi: 10.3389/fgene.2022.886781 (PMC9393255; doi:10.3389/fgene.2022.886781)
Supplement: Supplementary file 2 [file DataSheet1.ZIP › 4dpi_KEGG.Gsea.1625100298584/KEGG_CYTOSOLIC_DNA_SENSING_PATHWAY.html]

Details for gene set KEGG\_CYTOSOLIC\_DNA\_SENSING\_PATHWAY[GSEA]

|  || Dataset | TMM\_4dpi\_gct\_format\_4dpi\_gct\_format.Class\_4dpi.cls #PC\_versus\_NC.Class\_4dpi.cls #PC\_versus\_NC\_repos |
| Phenotype | Class\_4dpi.cls#PC\_versus\_NC\_repos |
| Upregulated in class | 1 |
| GeneSet | KEGG\_CYTOSOLIC\_DNA\_SENSING\_PATHWAY |
| Enrichment Score (ES) | 0.58123106 |
| Normalized Enrichment Score (NES) | 1.7369657 |
| Nominal p-value | 0.007032349 |
| FDR q-value | 0.082701325 |
| FWER p-Value | 0.3294 |
Table: GSEA Results Summary

  

Fig 1: Enrichment plot: KEGG\_CYTOSOLIC\_DNA\_SENSING\_PATHWAY      
 Profile of the Running ES Score & Positions of GeneSet Members on the Rank Ordered List

  

| SYMBOL | TITLE | RANK IN GENE LIST | RANK METRIC SCORE | RUNNING ES | CORE ENRICHMENT || 1 | IRF7 | na | 85 | 1.706 | 0.1429 | Yes |
| 2 | CASP1 | na | 231 | 1.288 | 0.2441 | Yes |
| 3 | RIPK3 | na | 409 | 1.055 | 0.3220 | Yes |
| 4 | ADAR | na | 412 | 1.051 | 0.4143 | Yes |
| 5 | CCL5 | na | 438 | 1.026 | 0.5024 | Yes |
| 6 | IKBKE | na | 505 | 0.959 | 0.5812 | Yes |
| 7 | IL18 | na | 1198 | 0.604 | 0.5766 | No |
| 8 | CCL4 | na | 1945 | 0.433 | 0.5524 | No |
| 9 | POLR3A | na | 2318 | 0.373 | 0.5542 | No |
| 10 | IKBKB | na | 3506 | 0.220 | 0.4745 | No |
| 11 | POLR3D | na | 3731 | 0.195 | 0.4729 | No |
| 12 | RIPK1 | na | 4121 | 0.158 | 0.4544 | No |
| 13 | TBK1 | na | 4986 | 0.079 | 0.3892 | No |
| 14 | NFKBIA | na | 5648 | 0.021 | 0.3359 | No |
| 15 | RELA | na | 5979 | -0.005 | 0.3088 | No |
| 16 | POLR3B | na | 5986 | -0.005 | 0.3088 | No |
| 17 | MAVS | na | 6400 | -0.038 | 0.2776 | No |
| 18 | POLR1D | na | 6504 | -0.047 | 0.2731 | No |
| 19 | NFKB1 | na | 6765 | -0.068 | 0.2574 | No |
| 20 | POLR3H | na | 7428 | -0.129 | 0.2136 | No |
| 21 | NFKBIB | na | 8760 | -0.257 | 0.1251 | No |
| 22 | POLR3F | na | 9085 | -0.294 | 0.1239 | No |
| 23 | CHUK | na | 9400 | -0.336 | 0.1272 | No |
| 24 | POLR1C | na | 9981 | -0.411 | 0.1149 | No |
| 25 | IL1B | na | 11033 | -0.617 | 0.0815 | No |
Table: GSEA details [plain text format]

  

Fig 2: KEGG\_CYTOSOLIC\_DNA\_SENSING\_PATHWAY      
 Blue-Pink O' Gram in the Space of the Analyzed GeneSet

  

Fig 3: KEGG\_CYTOSOLIC\_DNA\_SENSING\_PATHWAY: Random ES distribution      
 Gene set null distribution of ES for **KEGG\_CYTOSOLIC\_DNA\_SENSING\_PATHWAY**
